# Common Clinical Characteristics and Rare Medical Problems of Fragile X Syndrome in Thai Patients and Review of the Literature

**DOI:** 10.1155/2017/9318346

**Published:** 2017-06-29

**Authors:** Chariyawan Charalsawadi, Juthamas Wirojanan, Somchit Jaruratanasirikul, Nichara Ruangdaraganon, Alan Geater, Pornprot Limprasert

**Affiliations:** ^1^Department of Pathology, Faculty of Medicine, Prince of Songkla University, Songkhla 90110, Thailand; ^2^Department of Pediatrics, Faculty of Medicine, Prince of Songkla University, Songkhla 90110, Thailand; ^3^Department of Pediatrics, Faculty of Medicine, Ramathibodi Hospital, Mahidol University, Bangkok 10400, Thailand; ^4^Epidemiology Unit, Faculty of Medicine, Prince of Songkla University, Songkhla 90110, Thailand

## Abstract

*Background. *Clinical characteristics of fragile X syndrome (FXS) have been well documented in Caucasians, whereas in Asians they have rarely been described. Those that have been conducted used small cohorts that utilized DNA for diagnosis and larger cohorts that utilized cytogenetics for diagnosis. This study is to describe clinical characteristics of FXS in a large cohort of Thai patients diagnosed by standard molecular methods.* Methods.* Seventy-seven index cases and 46 affected relatives diagnosed with FXS were recruited into the study. To determine frequencies of common characteristics of FXS in prepubertal boys, we reviewed 56 unrelated cases aged between 18 and 146 months. To list rare medical problems, we reviewed 75 cases aged between 8 months to 71 years old, including 53 index cases and 22 affected relatives. In addition, we selected 16 clinical studies from various ethnicities for comparison with our findings.* Results. *In prepubertal boys with FXS, attention deficit and/or hyperactivity, prominent ears, macroorchidism, and elongated face were observed in 96%, 80%, 53%, and 48% of patients, respectively, whereas recognizable X-linked inheritance presented in 11% of patients. IQ scores ranged between 30 and 64 (mean ± SD = 43 ± 9, *n* = 25). We observed clinical findings that rarely or have never been reported, for example, medulloblastoma and tetralogy of Fallot.* Conclusion. *Attention deficit and/or hyperactivity and prominent ear are the most common behavioral and physical features in prepubertal boys with FXS, respectively. There are differences in frequencies of clinical characteristics observed between ethnicities; however, it is difficult to draw a solid conclusion due to different recruitment criteria and sample sizes within each study.

## 1. Introduction

Fragile X syndrome (FXS; OMIM#300624) is one the most common causes of intellectual disability, with a prevalence of approximately 1 : 4,000 in males and 1 : 6,000 in females. The gene responsible for FXS,* fragile X mental retardation 1 (FMR1)* gene, is located on chromosome Xq27.3. FXS is associated with an unstable expansion of CGG trinucleotide repeats within the 5′untranslated region of the gene. Alleles with between 5 and 54 CGG repeats and between 55 and 200 CGG repeats are classified as normal and premutation (PM), respectively. A full mutation (FM) allele is defined as having more than 200 CGG repeats. Individuals with the FM allele exhibit a broad spectrum of clinical characteristics [1–5].

DNA testing for FXS is strongly recommended for all children with developmental delay of unknown causes. Most males with FXS have moderate intellectual disability [2, 3]. Classic features of FXS are elongated face, large and protruding ears, and macroorchidism. Other physical features and medical problems may include high-arched palate, strabismus, refractive error, frequent sinusitis and otitis, excessive joint laxity, hyperextensible metacarpophalangeal joints, double-jointed thumbs, flat or pronated feet, softness and smoothness of the skin, hand calluses, cardiovascular abnormalities (e.g., aortic root dilation, mitral valve prolapse), neurological involvement (e.g., hypotonia, motor incoordination, and seizure), gastrointestinal problems (e.g., gastroesophageal reflux, constipation, and loose bowel movements), obstructive sleep apneas, and unusual growth patterns (e.g., increased birth weight, macrocephaly). Behavioral features such as attention deficit, hyperactivity, autistic-like behaviors, shyness, social anxiety, tactile defensiveness, aggressiveness, sleep problems, and stereotypies (e.g., tics, hand mannerisms) are frequently observed among individuals with FXS [1–5]. Affected females normally have less clinically severe manifestations than affected males and the degree of such manifestations depends largely on the degree of X-inactivation of the abnormal X chromosome [4].

While information regarding the clinical characteristics of FXS has been well documented in Caucasians, it has rarely been described in Africans and Asians. In Thailand, prevalence of FXS among Thai boys with developmental delay was about 7% [35]. There is another earlier report on the clinical characteristics of FXS in a very small cohort of Thai patients with FXS [36]; however, a systematic review in a larger cohort of Thai patients has never been carried out. Previous studies in other groups of Asians have also been conducted in small cohorts of patients, ranging from 10 to 25 patients per study. However, these studies had only limited use because they either utilized molecular methods for diagnosis in small cohorts of patients or utilized cytogenetic method for diagnosis (Table 1). Over the years we have faced difficulties when providing genetics counseling, due to lack of current local information as the information regarding frequencies of clinical characteristics of FXS was from studies in Caucasian patients. The first aim of this study was to determine the frequencies of common clinical characteristics of Thai prepubertal boys with FXS. The second aim was to describe rare medical problems in a cohort of Thai patients with FXS. The third aim was to compare our findings with relevant studies from various world areas.

## 2. Methods

### 2.1. Study Participants

We reviewed a retrospective cohort of Thai patients diagnosed with FXS between April 1993 and June 2015. Inclusion criteria were patients with FXS who were diagnosed by molecular methods and had a 5-item clinical checklist [37] and/or medical record available. These patients were referred from multiple locations in Thailand to our institution (Faculty of Medicine, Prince of Songkla University), the leading diagnostic center for FXS in Thailand. A total of 123 Thai patients from 77 families, including 99 males and 24 females, were diagnosed with FXS during the study. These patients were from 11 hospitals, including 87 patients from our institutions (Faculty of Medicine, Prince of Songkla University and Faculty of Medicine, Ramathibodi Hospital, Mahidol University). All individuals diagnosed with FXS had over 200 CGG repeats. Of these, 11 males and 2 females were mosaic for FM and PM. After the physical examination, the checklists were filled out and sent to our laboratory along with the patient's blood specimens.

Upon receiving patient's blood specimens, we performed DNA analysis. Briefly, the lengths of CGG repeat segments were analyzed using either the modified nonradioactive PCR method [35] or fluorescent PCR fragment analysis. The methylation status of the* FMR1*gene was screened using methylation-specific PCR [38].* Eco*RI/*Eag*I double digestion and Southern blot analysis using the StB12.3 probe results were accepted as the gold standard for the diagnosis. We utilized the Southern blot analysis to confirm diagnosis in all affected cases and suspicious cases. Once the diagnosis of FXS was made in an index case, we contacted his/her family to further examine for FXS status in relatives and genetic counseling was given at that time.

### 2.2. Data Collection

This study utilized existing records and conducted record reviews. To determine frequencies of common clinical characteristics based on the 5-item clinical checklist in prepubertal boy with FXS, 56 unrelated prepubertal boys, who had the clinical checklist available, were reviewed. Of the 56 unrelated prepubertal boys with FXS, 11 boys were mosaic for FM and PM. At the time of diagnosis, all patients received a physical examination at our institutions, except 4 cases with only the 5-item clinical checklist and 3 cases with the 5-item clinical checklist and medical records were referred from other hospitals. The common clinical characteristics were measured by using standardized measures except for elongated face that was based on physician judgment.

The 5-item clinical checklist items included family history, elongated face, prominent and large ears, attention deficit and/or hyperactivity, and macroorchidism. Regarding inheritance pattern, we defined X-linked inheritance when at least two individuals with intellectual disabilities present in a family in more than one generation without a male to male transmission. Inheritance patterns were analyzed from an existing pedigree, which was taken by authors and other physicians at the time of the diagnosis. Family history included learning difficulties, developmental delay, and intellectual disabilities, which were recorded in the pedigree of at least 3 generations. The judgment of having an elongated face was primarily based on a clinical impression of a long jaw together with a high forehead. Prominent ears were diagnosed when the angle of the ear and the face was nearly 90 degrees and when the longest axis of the ears measurement was greater than the 95th percentile of the standard scale [39]. Macroorchidism was diagnosed when testicular volume measured with a Prader orchidometer was greater than the 95th percentile of the modified standard scale [39] (age ≤ 8 years, 1-2 ml: score = 0, 3 ml: score = 1, >3 ml: score = 2 or age >8 years, 95th percentile −2 ml: score = 0, 95th percentile ±1 ml: score = 1, 95th percentile +2 ml; score = 2). Attention deficit and hyperactivity were diagnosed according to the DSM-IV criteria and scored (none; score = 0, either attention deficit or hyperactivity; score = 1, both attention deficit and hyperactivity; score = 2). In addition, autistic-like behaviors were recorded when one of the following behaviors was presented: poor eye contact, tactile defensiveness, hand biting, hand flapping, and delayed or perseverative speech.

**Figure 1:**
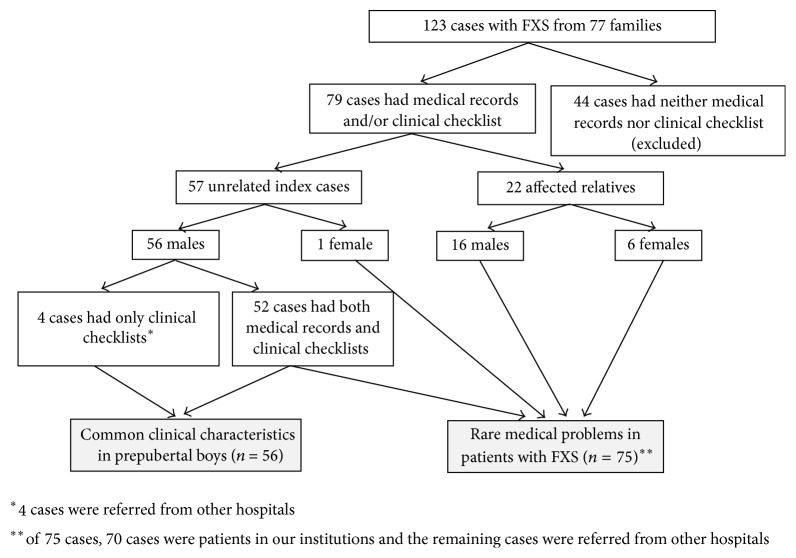
Diagram depicting summary of study design.

To list rare medical problems, we reviewed medical records of both unrelated patients with FXS and their affected relatives who had medical records available, including 53 unrelated index cases (52 males and 1 female) and 22 affected relatives (16 males and 6 females). Of these 75 cases, 70 cases were patients in our institutions and the remaining cases were referred from other hospitals (Figure 1).

Medical record data were extracted from patient history files and laboratory request forms. These data included chief compliant, age at diagnosis, intelligence quotient (IQ), developmental profile, behavioral patterns, and medical problems. Data extracted from the 5-item clinical checklist and medical records were recorded in Microsoft Excel.

In addition, we carried out a literature review on the topic of frequencies of clinical characteristics associated with FXS. We searched for relevant articles using PubMed with the primary keyword “fragile X syndrome,” as associated with the other keywords: clinical, characteristics, phenotype, population, and frequency. Only articles that were accessible and published in English were selected for the review.

### 2.3. Statistical Analysis

Logistic regression analysis was used to answer the question of which item in the 5-item clinical checklist was related to age at diagnosis. We grouped the 56 unrelated prepubertal boys into 2 groups, a group of boys diagnosed <8 years old (*n* = 37) and ≥8 years old (*n* = 18). For binary traits (presence/absence of characteristic), logistic regression analysis was also used to evaluate possible associations between ethnicity and the presence of the physical characteristics in the 5-item checklist.

The study was approved by the Research Ethics Committee (no. 58-239-05-1).

## 3. Results

### 3.1. Age of Diagnosis and Chief Complaint

The 56 unrelated prepubertal boys had been diagnosed between 18 and 146 months of age (mean ± SD = 75.3 ± 36.2). About 51% of the diagnoses were made when the children were between the ages of 5 and 10 and 33% at under 5 years of age. Only 22% of our patients were diagnosed with FXS when the child was under 36 months of age. The two most common chief complaints that led the parents/caregivers to seek medical attention were developmental delay and school problems, for example, learning difficulties and attention deficit.

### 3.2. Development and Intellectual Function

All prepubertal boys in the study had developmental delay, especially language delay. Language delay was warranted when children cannot speak any meaningful words at the age of 15 months and could not speak at least three meaningful words at the age of 18 months. The latter did not consider names of familiar people. Speech delay was determined when children could not speak two-word phrases and had vocabulary of less than 50 words or when children could not speak complete sentences, or 50% of speech could be understood by strangers at the age of 36 months. A total of 25 boys had IQ scores evaluated, either using the Stanford–Binet Intelligence Scale or the Wechsler Intelligence Scale for children. Their IQ scores ranged between 30 and 64 (mean ± SD = 43 ± 9). According to the ICD-10 classifications, about 70% had an IQ indicating a moderate range of intellectual disability (IQ between 35 and 49). Mild (IQ between 50 and 69) and severe (IQ between 20 and 34) intellectual disabilities were documented in 17% and 13% of the patients, respectively. Regarding IQ and mosaicism status, 2 mosaic patients had moderate (IQs of 38 and 40) and the other 2 mosaic patients had mild (IQs of 50 and 57) intellectual disabilities.

### 3.3. Common Clinical Characteristics According to the 5-Item Clinical Checklist

Out of the 54 families we studied, excluding an orphan patient and a patient with unknown family history, the X-linked pattern was observed in 6 families (11%). About 59% of the patients had no family history of intellectual disability. The remaining 30% of the patients had unclassified inheritance pattern.

The most common clinical characteristic in this cohort was attention deficit and/or hyperactivity, which was observed in 96% of the patients. Approximately 48% and 80% of the patients had an elongated face and prominent ears, respectively. Macroorchidism was observed in 53% of the patients (one of these patients who had unilateral macroorchidism that was apparent when he entered puberty was reported elsewhere [40]). It is interesting to note that 44 prepubertal boys, including affected relatives, were younger than 8 years old, of whom 52% and 9% had obvious and borderline macroorchidism, respectively. Number of prepubertal boys with FXS for each clinical item according to the 5-item clinical checklist is shown in Supplementary Table 1 (in Supplementary Material available online at https://doi.org/10.1155/2017/9318346).

The findings from our literature review regarding frequencies of clinical characteristics associated with FXS are shown in Table 1. In addition, other clinical characteristics recognized among the 56 unrelated boys including irritability (14%), hyperphagia (14%), hypertelorism (9%), thumb sucking (9%), hand flapping (9%), repetitive speech (9%), hand/nail biting (7%), talkative (7%), echolalia (5%), and toe-walking (5%) were observed.

Logistic regression analysis was used to answer the question of which item in the 5-item clinical checklist was related to age at diagnosis. We found that effects of family history, facial elongation, and macroorchidism on age at diagnosis were not significant. However, boys with attention deficit and/or hyperactivity and those with large and/or prominent ears were more likely to be diagnosed at age of younger than 8 years (OR = 5.4, 95% CI = 1.1–25.2; OR =5.9, 95% CI =1.4–24.8, resp.) (Table 2).

For binary traits (presence/absence of characteristic), we found that the presence of facial features (elongated face and large ears) and macroorchidism were significantly different among ethnic groups (Table 3).

### 3.4. Rare Medical Problems

Seizure was documented in 5 patients, excluding a patient with medulloblastoma. Among these five patients, one patient had 4 episodes of febrile seizure between the ages of one month and 5 years. For nonfebrile seizure patients, onset of seizure was between 2 years and 7 years of age and an electroencephalogram had been performed in 3 patients, with no definite abnormalities documented in 2 patients, and the other patient had had a tonic-clonic seizure with characteristic spike and sharp wave in the bilateral hemispheres. This latter patient was previously diagnosed with Sotos syndrome and pervasive developmental disorder. Other rare medical problems observed in this cohort are summarized in Table 4. We observed very unusual clinical findings in Thai patients with FXS that have never been reported in the literature, including tetralogy of Fallot and primary amenorrhea.

## 4. Discussion

### 4.1. FXS Diagnosis, Development, and Intellectual Functioning

In the era of molecular FXS diagnosis, FXS is still difficult to recognize and diagnose in all ethnic groups, which could be attributable to a lack of an obvious phenotype at birth and only subtle phenotypes during prepubescence. The mean age at diagnosis was 128.4 months (10.7 years) in a French study [41], 51.4 months (4.3 years) in a South Korean study [42], 35 to 37 months (2.9–3.1 years) in a US study [43], 66 months in Australian study [44], and 75 months (6.3 years) in our study. FXS is generally not diagnosed until developmental delay or learning difficulty becomes evident. Our study was in agreement with previous studies in that nearly all individuals with FXS have developmental delay, especially language delay [45]. Intellectual functioning as measured by standard psychological tests varies widely in individuals with FXS, ranging from average intelligence to severe intellectual disability. The variability of IQ scores is positively correlated with fragile X mental retardation protein status [4, 45]. Individuals with full methylation typically have IQ scores indicative of mild to moderate intellectual disability. For individuals with FXS mosaicism, the IQ scores normally indicate borderline to low average intelligence [4]. In our study, about 87% of the patients had IQ scores indicating a mild to moderate range of intellectual disability, regardless of mosaicism status. However, IQ measurement in individuals with FXS is challenging, partly due to a lack of tools sensitive enough for individuals with lower levels of cognitive functioning and also partly due to the fact that individuals with FXS tend to have attention problems which can limit their ability to complete certain aspects of any testing [45].

A family history of intellectual disability was observed in between 30% and 92% of patients in different studies (Table 1). Differences in observed frequencies may be influenced by different recruitment criteria and sample sizes. Given that FXS is X-linked inherited, one may expect the presence of multiple affected males in the family. Based on a study in “unrelated patients,” we found that it was uncommon to see X-linked inheritance. An interesting point here is that we observed ~60% of our patients without a family history of intellectual disabilities, implying that the absence of a family history of intellectual disability does not exclude the possibility of an FXS diagnosis.

### 4.2. Common Physical Characteristics

It is known that the physical hallmarks of FXS, including elongated face, large and protruding ears, and macroorchidism, are subtle during early childhood and normally only become prominent in early adolescence. In this study, elongated face was observed in about half of the patients, comparable to a study in Caucasian prepubertal boys [4]. However, a study in 34 young Caucasian Americans and 2 African Americans observed a much higher frequency of elongated face in prepubertal boys (83%) [6]. In East and South East Asians studies, the prevalence of elongated face was higher in a Korean (90%) [21] than in Thai (48%) prepubertal boys with FXS (*p* = 0.015). For Turkish prepubertal boys with FXS, elongated face was statistically different between studies (83% versus 29%, *p* < 0.001) [14, 15]. These studies indicate that even in young individuals with FXS, there is a wide variation of facial features among ethnicities and even within the same ethnicity. Logistic regression analysis showed that Americans FXS patients were more likely to have elongated face than Europeans and Indians, whereas no statistical differences were shown between Asians or Middle Eastern FXS patients and other ethnicities (Table 3). Prominent ears were the second most common features in our patients, and large ears were also very common. These features are quite common across ethnicities, though with some variation. In Caucasian with FXS, there is a wide variation in frequency of large ears, varying from 24% to 100%, while they have been noted in 46% to 90% in Asians with FXS. In young Saudis with FXS, large ears were observed in 30% of the patients, close to 29% found in young Turkish boys with FXS (Table 1). Sample size difference is one of the factors which could explain such differences in frequencies of facial features observed in FXS among studies. The differences could also be attributable to different methods used to define such characters, whether by anthropometric measurement or by visual impression. The latter tends to be biased, depending on the individual physician's experience. In addition, it is somewhat challenging to do anthropometric measurements in young children with FXS as they are habitually hyperactive and dislike being touched (i.e., tactile defensiveness).

Macroorchidism is present in over 80% of postpubertal males. A dramatic increase in the size of testicles has been seen in FXS boys between 8 and 10 years old, probably as a result of gonadotropin stimulation [4]. In our study, all postpubertal males had macroorchidism (data not shown) and about half of the prepubertal boys had it. Approximately 52% of prepubertal boys younger than 8 years old had testicular volumes greater than the 95th percentile, comparable with a study by Butler et al. 1991 [39], which found that more than 50% of boys with FXS had testicular volumes greater than the 95th percentile. Studies in males with FXS younger than 20 years old have found that macroorchidism was not very common in middle east countries (Turkey, Saudi Arabia, and Egypt), with frequencies of 11–23%, 15%, and 21%, respectively, while it was more common in Thais and Koreans, with frequencies of 53% and 70%, respectively [13–15, 17, 21]. For Americans with FXS younger than 20 years of age, macroorchidism was observed in frequencies ranging from 19% to 63% [6–8]. The difference between frequencies of macroorchidism in young individuals with FXS could be attributable to testicular volume's cut-off criterion. For example, while other studies used testicular volumes of greater than the 95th percentile or approximately >2.5 ml [4, 39], a less frequency of macroorchidism in one study used a testicular volume of >4 ml to define macroorchidism. The latter study observed that only 4% of boys with FXS at ages less than 7 years had significant macroorchidism; however, mean testicular volumes of those boys were significantly larger than boys without FXS at the same age. At 8 to 10 years old, all of their patients with FXS had testicular volume more than 3.75 ml [18].

### 4.3. Common Behavioral Characteristics

In this study, we found that hyperactivity and/or attention deficit were the most common symptoms for FXS, especially in young boys. Various studies have found that hyperactivity and/or attention deficit were common among both Caucasians and Asians with FXS (Table 1). Hyperactivity and/or attention deficit were observed as the most common features in FXS in some studies [15, 17, 21, 46]. Symptoms have been found to manifest even in high-functioning boys with FXS, although in such patients the symptoms usually improved with age [4].

Autistic-like behaviors such as hand flapping, hand biting, perseverative speech, shyness, and poor eye contact have been observed in a number of individuals with FXS; however, only 15–28% of them fulfilled the DSM-III-R or DSM-IV criteria for the diagnosis of autism [4]. In this study, autistic-like behaviors were described in 27% of unrelated patients; however, only two patients were diagnosed with autism before the* FMR1* DNA testing was performed.

Shyness and social anxiety are observed among individuals with FXS and in females more than males. In males, hyperactivity, attention deficit, and impulsivity are often more prominent than shyness and social anxiety. In this study, approximately 20% of the boys had shyness. Shyness is sometimes difficult to distinguish from autistic-like behavior; however, children with shyness are able to communicate. Shyness is observed when children attempt to avoid or have minimal eye contact and communication with strangers (physicians and nurses) but not with familiar people (parents and relatives). This number is less than that of Caucasians (58%) [7]. Aggressiveness is a more of a problem in adolescent men with FXS but is not usually an issue later in life [4]. We also observed aggressiveness in boys with FXS less often than reported in studies of Caucasians (Table 1).

### 4.4. Rare Medical Problems

Seizure is the most common neurologic abnormality in FXS. Incidence of seizures in FXS has been reported ranging from 4.4% to 20% [5, 47]. The characteristics of seizure reported in individuals with FXS have been variable. Tonic-clonic seizure, complex partial seizure, simple partial seizure, and febrile seizure have been reported in FXS, with status epilepticus only rarely reported. Partial seizures are more common than generalized seizures in FXS [4, 5]. In the current study, unknown cause of seizure was documented in 5 patients (~7%) and we found no specific characteristics of seizure and EGG pattern as mentioned previously.

Interestingly, we found a patient who presented with seizure. Upon investigation, medulloblastoma was diagnosed in this patient. Medulloblastoma is a posterior fossa tumor and is the most common malignant childhood brain tumor. Occurrence of medulloblastoma has rarely been reported in individuals with FXS (Table 4). It was suggested that FMRP may be related to oncogenesis of medulloblastoma through a loss of FMRP resulting in reduced *β*-catenin levels and a defective Wnt signaling pathway, where both pathways have been implicated in medulloblastoma oncogenesis [23]. Other brain tumors have rarely been reported in FXS. Kalkunte et al. [2007] reported a 10-year-old boy with FXS and inoperable midbrain glioblastoma, who was still alive 8 years after diagnosis [48].

Cardiac involvement in FXS usually presents with mitral valve prolapse and aortic root dilatation, which are more prevalent in adults than children with FXS. The prevalence of mitral valve prolapse and aortic root dilatation in children was lower at about 6% [9]. A recent study showed less frequency of mitral valve prolapse at birth to 55 years old at about 0.8% [5]. In the current study, we did not see FXS individuals with mitral valve prolapse and/or aortic root dilatation. We, herein, had seen the patient of a boy with FXS who had a more complicated cardiac defect, and, to the best of our knowledge, we are the first to report this particular conotruncal heart defect in FXS. Nonetheless, we are aware that our observation could be a coincidental finding. Tetralogy of Fallot is recognized in certain genetic syndromes, including 22q11.2 deletion and trisomy 21. Mutations in* NKX2-5* (5q35.1),* GATA4* (8q23.1),* ZFPM2* (8q23.1),* GATA6* (18q11.2),* GDF1* (19p13.11),* JAG1* (20p12.2), and* TBX1* (22q11.21) have been reported in sporadic cases with tetralogy of Fallot; however, interaction of these genes and* FMR1* gene has never been reported [49]. In addition, one characteristic we found in our study has never been reported elsewhere, at least to the best of our knowledge, which is primary amenorrhea in females with FM, although this may be a coincidental finding.

## 5. Conclusion

Our study provides insight into the clinical characteristics of FXS in Thai patients and in comparison to other ethnicities, which is necessary for genetic counseling. Our study has limitations as it was a retrospective review from available medical and laboratory records. Furthermore, some patients were lost to follow-up after the diagnosis was made. It is also difficult to draw solid conclusions with respect to differences in frequencies of clinical characteristics observed between ethnicities, partly due to different recruitment criteria and sample sizes. Our review is at least adding to the knowledge on FXS and may be useful in further studies.

## Supplementary Material

Supplementary Table 1. Number of prepubertal boys with FXS for each clinical item, according to the 5-item clinical checklist.

## Figures and Tables

**Table 1:** Common clinical characteristics observed in patients with FXS.

Region	America						Europe		Middle East					South Asia			East and South East Asia	
Reference	Lachiewicz et al. 2000 [6]^a^	Giangreco et al. 1996 [7]	Merenstein et al. 1996 [8]^b^	Merenstein et al. 1996 [8]^b^	Crabbe et al. 1993 [9]^c^	Butler et al. 1991 [10]^d^	de Vries et al. 1999 [11]^e^	Arvio et al. 1997 [12]	Behery 2008 [13]	Alanay et al. 2007 [14]^f^	Demirhan et al. 2003 [15]	Bastaki et al. 2004 [16]^g^	Iqbal et al. 2000 [17]^h^	Kanwal et al. 2015 [18]^i^	Guruju et al. 2009 [19]^j^	Verma and Elango 1994 [20]^k^	H. R. Moon and S. Y. Moon 1993 [21]	Our study^l^
*Physical Characteristics*																		
Elongated face	83%	33%	62%	79%	NA	NA	51%	NA	35%	83%	29%	100%	NA	38%	32%	60%	90%	48%
Large ear (L)/prominent ears (P)/both (B)	72% (L)	83% (L)	24% (L) 78% (P)	45% (L) 54% (P)	100% (L)	95% (L)	27% (B)	84% (L)	53% (B)	90% (L/P)	43% (P) 29% (L)	90% (L)	30% (L)	46% (L/P)	88% (L)	60% (L/P)	90% (L)	59% (L) 80% (P) 49% (B)
Macroorchidism	63%	NA	54%	91%	19%	84%	59%	89%	21%	23%	11%	55%	15%	NA	94%	30%	70%	53%
High-arched palate	94%	NA	50%	53%	63%	NA	NA	NA	NA	NA	21%	100%	NA	NA	NA	20%	NA	21%
Flat feet	69%	NA	82%	54%	NA	NA	NA	NA	NA	NA	NA	30%	NA	NA	NA	NA	NA	21%
Hyperextensible joint	100%	NA	82%	46%	82%	58%	41%	57%	47%	76%	7%	100%	NA	15%	68%	NA	NA	38%

*Behavioral Characteristics*																		
Hyperactivity (H)/attention deficit (A)/both (B)	NA	59% (B) 8% (H)	89% (H)	69% (H)	NA	63% (A) 74% (H)	NA	57% (B)	44% (H)	23% (B) 87% (A) 83% (H)	71% (A) 93% (H)	85% (B)	92% (H)	69% (B)	80% (A) 84% (H)	70% (H)	80% (A) 90% (H)	75% (B) 93% (A) 85% (H)
Autism/autistic-like	NA	50%	NA	NA	NA	NA	NA	NA	15%	32%	29%	45%	0%	NA	NA	NA	60%	27%
Shyness	NA	NA	58%	73%	NA	NA	NA	68%	NA	NA	NA	NA	NA	NA	NA	NA	30%	20%
Aggressiveness	NA	NA	59%	54%	NA	NA	NA	NA	NA	NA	36%	NA	NA	NA	NA	NA	NA	21%

*Study Participants*																		
FH of ID	69%	59%	NA	NA	NA	74%	78%	30%	68%	66%	50%	70%	46%	46%	92%	NA	NA	41%
Diagnostic methods	CG, DNA	CG, SB	PCR, SB	PCR, SB	CG	CG	PCR, SB	CG	RT-PCR	SB	CG	CG, PCR	CG	PCR, SB	PCR, SB	CG	CG	PCR, SB
Sample size (male : female)	36 : 0	11 : 1	125 : 0	93 : 0	16 : 1	19 : 0	59 : 0	20 : 0	34 : 0	103 : 0	9 : 5	20 : 0	24 : 2	10 : 3	25 : 0	20	10 : 0	56 : 0
Age range (years)	NA	1.5–33	3–12	>12	4–13	3.7–71.9	NA	21–54	2–20	2–22	2–12	Pre puberty (45%) Post puberty (55%)	NA	NA	4–12 years (20%) 12–>16 years (80%)	<10 years (65%) >10 years (35%)	2–10.2	1.5–12.2
Mean age (±SD) (years)	6.2 (±2.4)	7.9	6.4 (±3.0)	22.4 (±8.7)	8.3	21.3	NA	31.7 (±11.0)	NA	7.2 (±4.0)	6.6 (±3.2)	NA	NA	NA	NA	NA	6.2 (±2.6)	6.3 (±3.0)

Country	USA	USA	USA	USA	USA	USA	Nether-lands	Finland	Egypt	Turkey	Turkey	Kuwait	Saudi Arabia	Pakistan	India	India	South Korea	Thailand

^a^Data from 34 Caucasians and 2 African Americans. ^b^Combined data from males with fully methylation, males with partial methylation, and males with mosaic FM/PM. Mean age and sample size for prepubertal males in fully methylation group, partial methylation group, and mosaic group were 6.4 ± 3 (*n* = 96), 6.4 ± 3 (*n* = 5), and 6.9 ± 3.1 (*n* = 4), respectively. Mean age and sample size for postpubertal males in fully methylation group, partial methylation group, and mosaic group were 22.4 ± 8.7 (*n* = 64), 22.1 ± 7.5 (*n* = 7), and 22.6 ± 12.1 (*n* = 22), respectively. Mean age showed in the table was from fully methylation group. ^c^Data from 14 Caucasians and 3 African Americans. ^d^Data from 15 Caucasians and 4 African Americans. ^e^Hyperextensible metacarpophalangeal digit V was found in 41% of patients and digits V and I in 15% of patients. Macroorchidism for both sides was 59% and for one side or moderate was 9%. ^f^Macroorchidism in prepubertal and postpubertal patients were 23% and 41%, respectively. Pervasive developmental disorder found in 32% of patients. ^g^85% of patients had sibling(s) with ID, and 70% of patients had relative(s) with ID. ^h^95.6% of population, including those with and without FXS, were younger than 20 years old. Of these, 83.4% were 5–15 years old. ^i^Age of all participants (including FXS and non-FXS patients) ranged from 4 to 40 years; mean ± SD 14.3 ± 7.0 years. ^j^Age of participants ranged from 4 to 8 years (4%), 8 to 12 years (16%), 12 to 16 years (12%), and >16 years (68%). ^k^Number of males and females was not specified. ^l^Family history of X-linked ID was 11%. Attention deficit and/or hyperactivity were 96%; NA = not available, FH = family history, ID = intellectual disability, PCR = polymerase chain reaction, SB = Southern blot, and CG = cytogenetics.

**Table 2:** Odds ratio showing the effect of clinical items in the 5-item clinical checklist on the likelihood that boys were diagnosed before 8 years old. Clinical items with no statistic difference (*p* value > 0.05) are not shown.

Clinical item	Odds ratio (95% confidence interval)	*p* value^*∗*^
Attention deficit/hyperactivity	5.38 (1.15–25.22)	0.033
Prominent/large ear	5.94 (1.42–24.79)	0.015

^*∗*^Wald test.

**Table 3:** Logistic regression analysis showing relationship between ethnicity and main physical characteristics, regardless of pubertal status. Odds ratio and 95% confidence interval for each characteristic are shown.

Ethnicity	Elongated face	Large ear	Macroorchidism
American	1^a^ (*n* = 266)	1^a^ (*n* = 302)	1^a^ (*n* = 290)
European (Finn and Dutch)	0.45^b^ (0.27–0.75) (*n* = 59)	4.45^b^ (1.80–10.97) (*n* = 20)	0.95^a^ (0.31–2.92) (*n* = 79)
Middle Easterner (Turkish, Kuwaiti, Saudi Arabian, and Egyptian)	1.06^ab^ (0.32–3.53) (*n* = 171)	1.11^a^ (0.22–5.60) (*n* = 60)	0.16^b^ (0.06–0.44) (*n* = 197)
South Asian (Indian and Pakistani)	0.33^b^ (0.15–0.74) (*n* = 58)	3.44^ab^ (0.84–14.00) (*n* = 45)	0.93^ab^ (0.11–7.93) (*n* = 45)
East and South East Asian (Korean and Thai)	0.53^ab^ (0.43–1.17) (*n* = 66)	1.95^ab^ (0.69–5.45) (*n* = 66)	0.63^a^ (0.24–1.66) (*n* = 66)
*p* value^*∗*^	*0.011*	*<0.001*	*<0.001*

Values within columns not having a superscript in common differ significantly (*p* < 0.05); ^*∗*^Wald test.

**Table 4:** Rare medical problems observed in patients with FXS in this study and previous reports.

Medical problem	Number of patients in our study	Previous patient report(s)
Tetralogy of Fallot	1^a^	(i) No previous report in PubMed

Medulloblastoma	1^b^	(i) Garrè et al. 2009 [22] reported an 18-month-old boy with FXS and medulloblastoma with extensive nodularity (ii) Alexiou et al. 2012 [23] reported an 11-year-old boy with methylation mosaicism for FXS and medulloblastoma

Sotos syndrome-like	2	(i) Beemer et al. 1986 [24] reported 2 boys with overgrowth, macrocephaly, minor facial anomalies and mild retardation, who were clinically diagnosed as Sotos syndrome and cytogenetic analysis results revealed fra(X)(q27)

Prader-Willi syndrome-like	3	(i) de Vries et al. 1993 [25] reported a patient with FXS when analyzing the DNA of 26 patients with suspected Prader-Willi syndrome but no abnormalities of chromosome 15. The authors also described 8 other patients with FXS and a Prader-Willi syndrome-like phenotype

Esotropia	1	(i) Hatton et al. 1998 [26] reported 8% of boys with FXS with strabismus

Astigmatism and hypermetropia	1	(i) Hatton et al. 1998 [26] reported 17% of boys with FXS with primarily hyperopia and astigmatism

Bilateral ptosis	1	Ptosis was described in previous literature [4]

Hypothyroidism	1	(i) Bregman et al. 1990 [27] reported blunted TSH response to thyrotropin releasing hormone in FXS subjects, suggesting subtle dysfunction within the hypothalamic-pituitary-thyroid axis

Bilateral conductive hearing loss	1^c^	(i) Alanay et al. 2007 [14] reported 5 males with FXS and minimal conductive hearing loss

Cleft palate	1^c^	Cleft palate was described in previous literature [4, 5]. It was also reported in patients with other anomalies: (i) Loesch et al. 1992 [28] reported a fragile X family with cleft lip and palate, digital and facial anomalies, and epilepsy (ii) Giampietro et al. 1996 [29] reported a 5-year-old boy with bilateral cleft lip and palate, ventricular septal defect, and a hypoplastic thumb

Depressive disorder	1^d^	(i) Tranebjærg and Ørum 1991 [30] reported 3 adult males with FXS and major depressive disorder

Precocious puberty	1^e^	(i) Butler and Najjar 1988 [31] reported an 8 1/2-year-old girl with precocious puberty and a unilateral ovarian cyst (ii) Moore et al. 1990 [32] reported a 2.8-year-old girl with early breast development, enlarged uterus and ovaries, and changes of hormonal profiles associated with true precocious puberty (iii) Kowalczyk et al. 1996 [33] reported a 10-year and 10-month-old girl with precocious puberty

Primary amenorrhea	1^f^	(i) No previous report in PubMed

Rectal cancer	1^g^	(i) Phelan et al. 1988 [34] reported mucin-producing adenocarcinoma of the colon in a 14-year-old male with FXS

^a^A 6-year-and-6-month-old boy had tetralogy of Fallot and was a mosaic for FM and PM, ^b^An 8-year-old boy had medulloblastoma with neuronal and glial differentiation, consistent with WHO grade IV, ^c^A 6-year-8-month-old boy had bilateral conductive hearing loss and cleft palate, ^d^A male was diagnosed with FXS at 11 years old and was suspected of depressive disorder at 23 years old, ^e^A 1-year-old girl had breast development (Tanner III) and mild coarse facies, ^f^A 40-year-old female had severe mental retardation, obesity, mild coarse facies, and primary amenorrhea. Her karyotype was 46,XX. No other investigation such as hormonal study to identify cause of primary amenorrhea, ^g^A 72-year-old female was diagnosed with adenocarcinoma of the rectum at 71 years of age.

## References

[1] OMIM OMIM entry - # 300624 - Fragile X syndrome. https://www.omim.org/entry/300624.

[2] Saul R. A., Tarleton J. C., Pagon R. A., Adam M. P., Ardinger H. H. (1993). FMR1-related disorders. *GeneReviews(®)*.

[3] Hersh J. H., Saul R. A., Saal H. M. (2011). Clinical report-health supervision for children with fragile X syndrome. *Pediatrics*.

[4] Hagerman R. J., Hagerman P. J. (2002). *Fragile X Syndrome: Diagnosis, Treatment, and Research*.

[5] Kidd S. A., Lachiewicz A., Barbouth D. (2014). Fragile X syndrome: a review of associated medical problems. *Pediatrics*.

[6] Lachiewicz A. M., Dawson D. V., Spiridigliozzi G. A. (2000). Physical characteristics of young boys with fragile X syndrome: reasons for difficulties in making a diagnosis in young males. *American Journal of Medical Genetics*.

[7] Giangreco C. A., Steele M. W., Aston C. E., Cummins J. H., Wenger S. L. (1996). A simplified six-item checklist for screening for fragile X syndrome in the pediatric population. *Journal of Pediatrics*.

[8] Merenstein S. A., Sobesky W. E., Taylor A. K., Riddle J. E., Tran H. X., Hagerman R. J. (1996). Molecular-clinical correlations in males with an expanded FMR1 mutation. *American Journal of Medical Genetics*.

[9] Crabbe L. S., Bensky A. S., Hornstein L., Schwartz D. C. (1993). Cardiovascular abnormalities in children with fragile X syndrome. *Pediatrics*.

[10] Butler M. G., Mangrum T., Gupta R., Singh D. N. (1991). A 15‐item checklist for screening mentally retarded males for the fragile X syndrome. *Clinical Genetics*.

[11] de Vries B. B., Mohkamsing S., van den Ouweland A. M. W. (1999). Screening for the fragile X syndrome among the mentally retarded: a clinical study. the collaborative fragile X study group. *Journal of Medical Genetics*.

[12] Arvio M., Peippo M., Simola K. O. J. (1997). Applicability of a checklist for clinical screening of the fragile X syndrome. *Clinical Genetics*.

[13] Behery A. K. (2008). Fragile x-syndrome: clinical and molecular studies. *The Journal of the Egyptian Public Health Association*.

[14] Alanay Y., Ünal F., Turanli G. (2007). A multidisciplinary approach to the management of individuals with fragile X syndrome. *Journal of Intellectual Disability Research*.

[15] Demirhan O., Taştemir D., Diler R. S., Firat S., Avci A. (2003). A cytogenetic study in 120 Turkish children with intellectual disability and characteristics of fragile X syndrome. *Yonsei Medical Journal*.

[16] Bastaki L. A., Hegazy F., Al-Heneidi M. M., Turki N., Azab A. S., Naguib K. K. (2004). Fragile X syndrome: a clinico-genetic study of mentally retarded patients in Kuwait. *Eastern Mediterranean Health Journal*.

[17] Iqbal M. A., Sakati N., Nester M., Ozand P. (2000). Cytogenetic diagnosis of fragile X syndrome: study of 305 suspected cases in Saudi Arabia. *Annals of Saudi Medicine*.

[18] Kanwal M., Alyas S., Afzal M. (2015). Molecular diagnosis of fragile X syndrome in subjects with intellectual disability of unknown origin: implications of its prevalence in regional Pakistan. *PLoS ONE*.

[19] Guruju M. R., Lavanya K., Thelma B. K. (2009). Assessment of a clinical checklist in the diagnosis of fragile X syndrome in India. *Journal of Clinical Neuroscience*.

[20] Verma I. C., Elango R. (1994). Variable expression of clinical features of Martin Bell syndrome in younger patients.. *Indian Pediatrics*.

[21] Moon H. R., Moon S. Y. (1993). Fragile site X chromosomes in mentally retarded boys.. *Journal of Korean medical science*.

[22] Garrè M. L., Cama A., Bagnasco F. (2009). Medulloblastoma variants: age-dependent occurrence and relation to gorlin syndrome-a new clinical perspective. *Clinical Cancer Research*.

[23] Alexiou G. A., Siozos G., Stefanaki K. (2012). Medulloblastoma in a child with fragile X syndrome. *Neuropediatrics*.

[24] Beemer F. A., Veenema H., De Pater J. M. (1986). Cerebral gigantism (Sotos syndrome) in two patients with fra(X) chromosomes. *American Journal of Medical Genetics*.

[25] De Vries B. B. A., Fryns J.-P., Butler M. G. (1993). Clinical and molecular studies in fragile X patients with a Prader-Willi-like phenotype. *Journal of Medical Genetics*.

[26] Hatton D. D., Buckley E., Lachiewicz A., Roberts J. (1998). Ocular status of boys with fragile X syndrome: a prospective study. *Journal of American Association for Pediatric Ophthalmology and Strabismus*.

[27] Bregman J. D., Leckman J. F., Ort S. I. (1990). Thyroid function in fragile-X syndrome males. *Yale Journal of Biology and Medicine*.

[28] Loesch D. Z., Hay D. A., Sheffield L. J. (1992). Fragile X family with unusual digital and facial abnormalities, cleft lip and palate, and epilepsy. *American Journal of Medical Genetics*.

[29] Giampietro P. F., Haas B. R., Lipper E. (1996). Fragile X syndrome in two siblings with major congenital malformations. *American Journal of Medical Genetics*.

[30] Tranebjærg L., Ørum A. (1991). Major depressive disorder as a prominent but underestimated feature of fragile X syndrome. *Comprehensive Psychiatry*.

[31] Butler M. G., Najjar J. L. (1988). Do some patients with fragile X syndrome have precocious puberty?. *American Journal of Medical Genetics*.

[32] Moore P. S. J., Chudley A. E., Winter J. S. D. (1990). Brief clinical report: True precocious puberty in a girl with the fragile X syndrome. *American Journal of Medical Genetics*.

[33] Kowalczyk C. L., Schroeder E., Pratt V., Conard J., Wright K., Feldman G. L. (1997). An association between precocious puberty and fragile X syndrome?. *Journal of Pediatric and Adolescent Gynecology*.

[34] Phelan M. C., Stevenson R. E., Collins J. L., Trent H. E. (1988). Fragile X syndrome and neoplasia. *American Journal of Medical Genetics*.

[35] Limprasert P., Ruangdaraganon N., Sura T., Vasiknanonte P., Jinorose U. (1999). Molecular screening for fragile X syndrome in Thailand. *The Southeast Asian Journal of Tropical Medicine and Public Health*.

[36] Ruangdaraganon N., Sura T., Sriwongpanich N., Limprasert P., Sombuntham T., Kotchabhakdi N. (2000). Prevalence and clinical characteristics of fragile X syndrome at child development clinic, Ramathibodi Hospital. *Journal of the Medical Association of Thailand*.

[37] Limprasert P., Vasiknanonte P., Jaruratanasirikul S. (2000). A clinical checklist for fragile X syndrome: screening of Thai boys with developmental delay of unknown cause. *Journal of the Medical Association of Thailand*.

[38] Charalsawadi C., Sripo T., Limprasert P. (2005). Multiplex methylation specific PCR analysis of fragile X syndrome: experience in Songklanagarind Hospital. *Journal of the Medical Association of Thailand*.

[39] Butler M. G., Allen G. A., Haynes J. L., Singh D. N., Watson M. S., Breg W. R. (1991). Anthropometric comparison of mentally retarded males with and without the fragile X syndrome. *American Journal of Medical Genetics*.

[40] Limprasert P., Jaruratanasirikul S., Vasiknanonte P. (2000). Unilateral macroorchidism in fragile X syndrome. *American Journal of Medical Genetics*.

[41] Biancalana V., Beldjord C., Taillandier A. (2004). Five years of molecular diagnosis of fragile X syndrome (1997–2001): a collaborative study reporting 95% of the activity in France. *American Journal of Medical Genetics*.

[42] Yim S.-Y., Bo H. J., Yang J. A., Kim H. J. (2008). Fragile X syndrome in Korea: a case series and a review of the literature. *Journal of Korean Medical Science*.

[43] Bailey D. B., Raspa M., Bishop E., Holiday D. (2009). No change in the age of diagnosis for fragile X syndrome: findings from a national parent survey. *Pediatrics*.

[44] Cotter M., Archibald A. D., Mcclaren B. J. (2016). Clinical audit of genetic testing and referral patterns for fragile X and associated conditions. *American Journal of Medical Genetics, Part A*.

[45] Huddleston L. B., Visootsak J., Sherman S. L. (2014). Cognitive aspects of Fragile X syndrome. *Wiley Interdisciplinary Reviews: Cognitive Science*.

[46] Lachiewicz A. M., Dawson D. V. (1994). Do young boys with fragile X syndrome have macroorchidism?. *Pediatrics*.

[47] Heard T. T., Ramgopal S., Picker J., Lincoln S. A., Rotenberg A., Kothare S. V. (2014). EEG abnormalities and seizures in genetically diagnosed fragile X syndrome. *International Journal of Developmental Neuroscience*.

[48] Kalkunte R., Macarthur D., Morton R. (2007). Glioblastoma in a boy with fragile X: an unusual case of neuroprotection. *Archives of Disease in Childhood*.

[49] OMIM OMIM entry - # 187500 - Tetralogy of Fallot; TOF. http://www.omim.org/entry/187500.

