# Supplementary material for: Common Clinical Characteristics and Rare Medical Problems of Fragile X Syndrome in Thai Patients and Review of the Literature
Source: Int J Pediatr. 2017 Jun 29;2017:9318346. doi: 10.1155/2017/9318346 (PMC5511659; doi:10.1155/2017/9318346)
Supplement: Supplementary file 1 — Supplementary Table 1. Number of prepubertal boys with FXS for each clinical item, according to the 5-item clinical checklist. [file 9318346.f1.docx]

**Supplementary Table 1. Number of prepubertal boys with FXS for each clinical item, according to the 5-item clinical checklist**

| Score | Number of patients for each clinical item | | | | | | | | | |
| --- | --- | --- | --- | --- | --- | --- | --- | --- | --- | --- |
|  | Family history | | Elongated face | | Prominent/ Large Ear | | Attention Deficit/ Hyperactivity | | Macroorchidism | |
| 0 | None | 32 | None | 13 | None | 6 | None | 2 | None | 20 |
| 1 | Unidentified | 16 | Borderline | 16 | Either prominent or large ears | 22 | Either attention deficit or hyperactivity | 11 | Borderline | 4 |
| 2 | X-linked | 6 | Present | 27 | Both prominent and large ears | 27 | Both attention deficit and hyperactivity | 38 | Present | 27 |
| Total | | 54 |  | 56 |  | 55 |  | 51 |  | 51 |
| Missing data | | 2 |  | 0 |  | 1 |  | 5 |  | 5 |
